# Supplementary material for: Determination of the Minimum Sample Amount for Capillary Electrophoresis-Fourier Transform Mass Spectrometry (CE-FTMS)-Based Metabolomics of Colorectal Cancer Biopsies
Source: Biomedicines. 2023 Jun 13;11(6):1706. doi: 10.3390/biomedicines11061706 (PMC10296550; doi:10.3390/biomedicines11061706)

Figure S1: TCA cycle with glutamine and glutamate. Blue and red bars represent nontumor and tumor sites, respectively. It was suggested that glutamine to glutamate production was enhanced in tumor sites compared to nontumor sites.

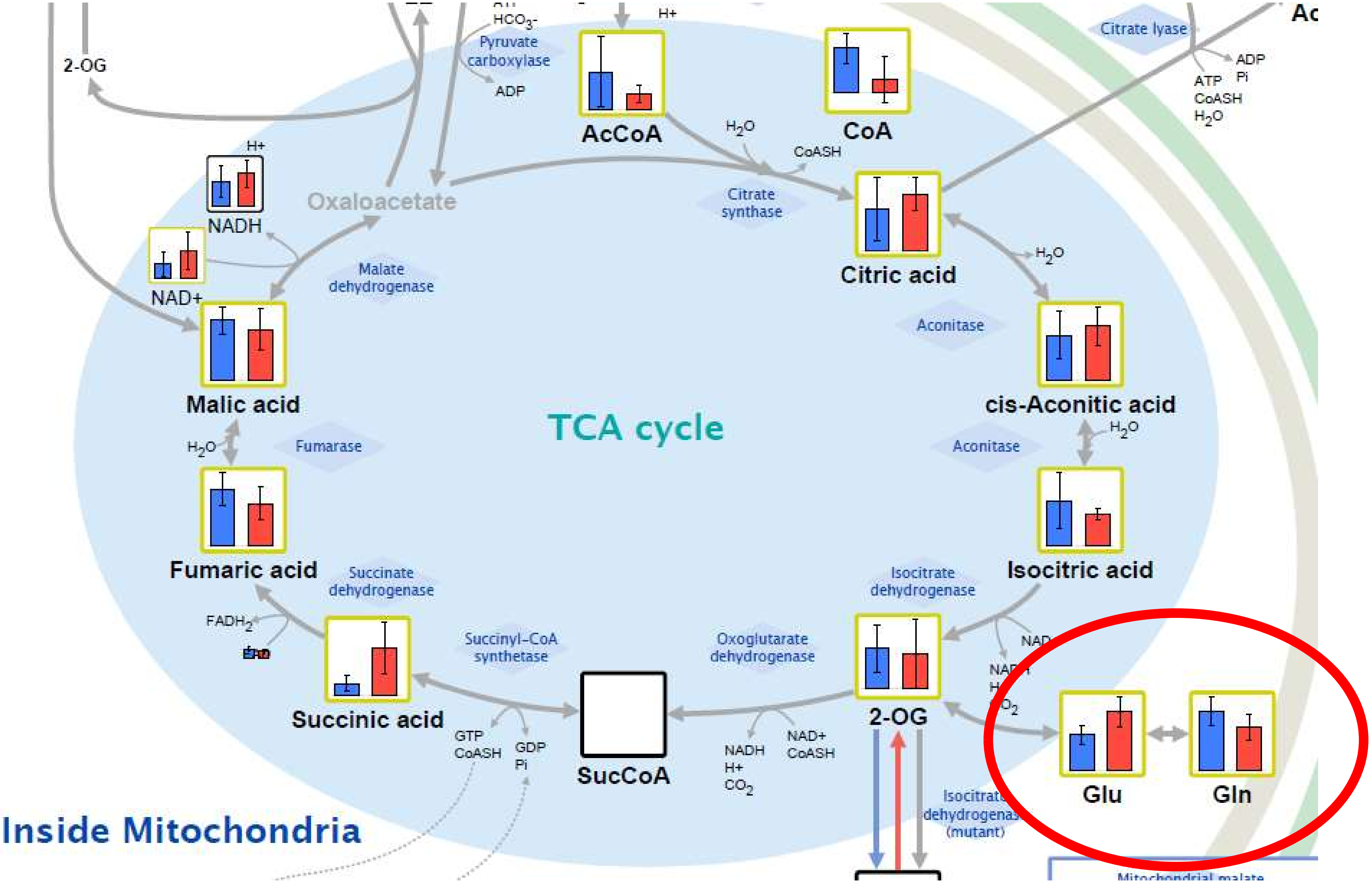

Supplement: Supplementary file 1 [file biomedicines-11-01706-s001.zip › Supplementary Materials/Figure S1.pdf]
