# Supplementary material for: Determination of the Minimum Sample Amount for Capillary Electrophoresis-Fourier Transform Mass Spectrometry (CE-FTMS)-Based Metabolomics of Colorectal Cancer Biopsies
Source: Biomedicines. 2023 Jun 13;11(6):1706. doi: 10.3390/biomedicines11061706 (PMC10296550; doi:10.3390/biomedicines11061706)

Figure S2: Purine metabolism. Blue and red bars represent nontumor and tumor sites, respectively. AMP and GMP were significantly increased at the tumor site presumably due to increased energy consumption and enhanced purine salvage pathway.

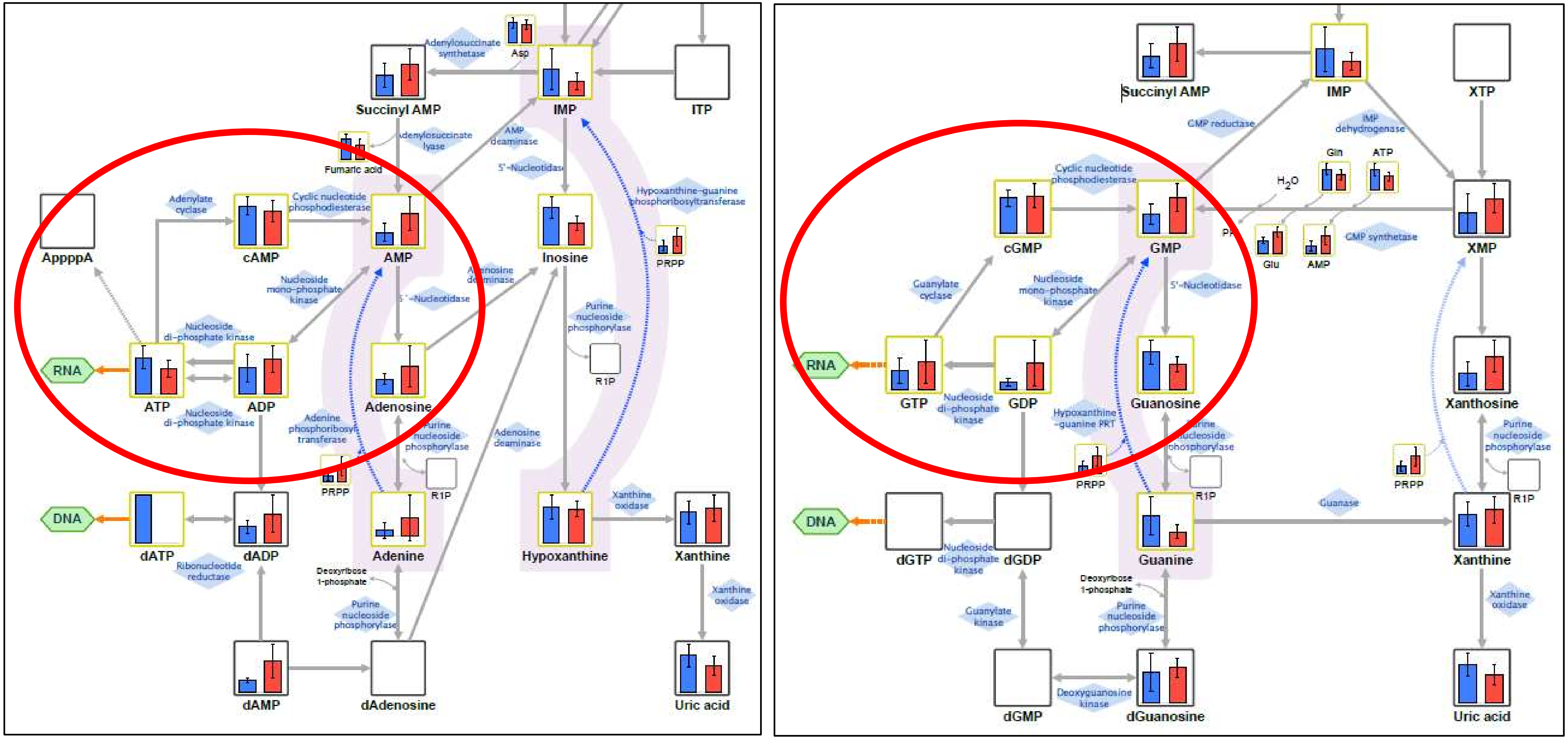

Supplement: Supplementary file 1 [file biomedicines-11-01706-s001.zip › Supplementary Materials/Figure S2.pdf]
