# Supplementary material for: Determination of the Minimum Sample Amount for Capillary Electrophoresis-Fourier Transform Mass Spectrometry (CE-FTMS)-Based Metabolomics of Colorectal Cancer Biopsies
Source: Biomedicines. 2023 Jun 13;11(6):1706. doi: 10.3390/biomedicines11061706 (PMC10296550; doi:10.3390/biomedicines11061706)

Figure S4: Methionine metabolism. Blue and red bars represent nontumor and tumor sites, respectively. Cysteine to cystine production was significantly enhanced at tumor sites. This was thought to be due to strong oxidative stress at the tumor site.

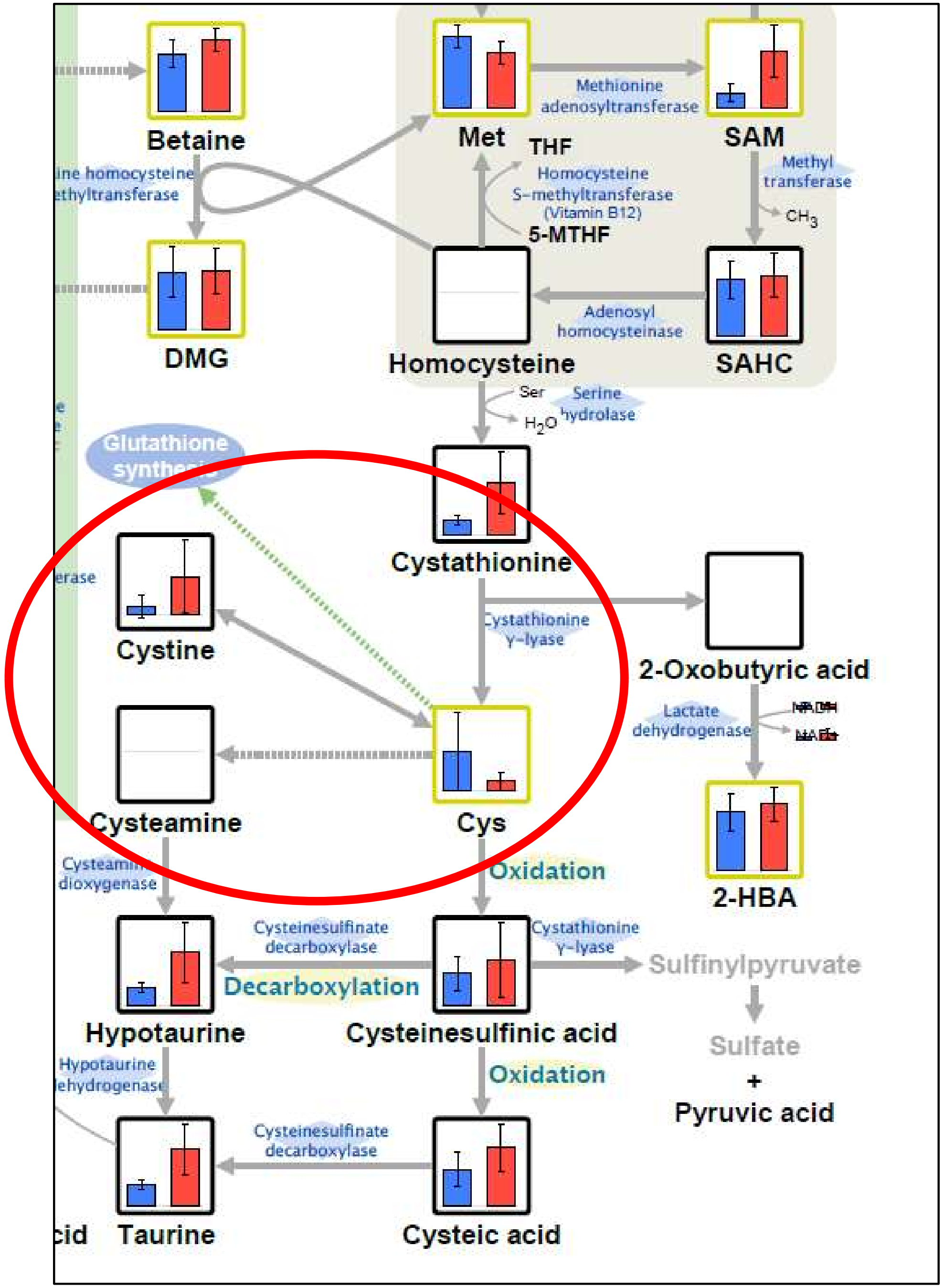

Supplement: Supplementary file 1 [file biomedicines-11-01706-s001.zip › Supplementary Materials/Figure S4.pdf]
