# Supplementary material for: Determination of the Minimum Sample Amount for Capillary Electrophoresis-Fourier Transform Mass Spectrometry (CE-FTMS)-Based Metabolomics of Colorectal Cancer Biopsies
Source: Biomedicines. 2023 Jun 13;11(6):1706. doi: 10.3390/biomedicines11061706 (PMC10296550; doi:10.3390/biomedicines11061706)

Figure S3: Pyrimidine metabolism. Blue and red bars represent nontumor and tumor sites, respectively. It was suggested that RNA synthesis and purine metabolism were enhanced in tumor sites compared to nontumor sites.

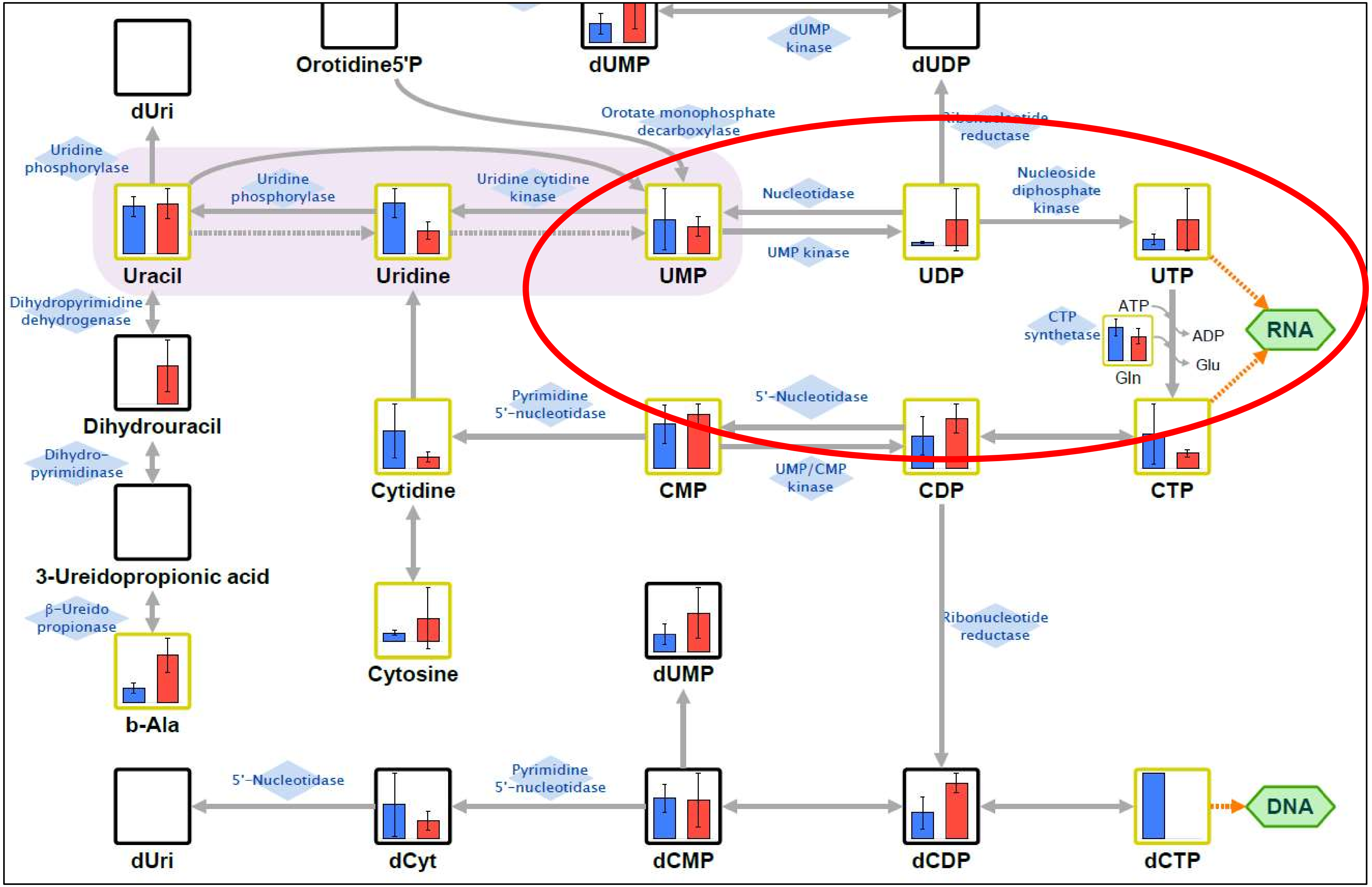

Supplement: Supplementary file 1 [file Figure S3.pdf › Supplementary Materials/Figure S3.pdf]
